# Modern Biodegradable Plastics—Processing and Properties Part II

**DOI:** 10.3390/ma14102523

**Published:** 2021-05-12

**Authors:** Janusz W. Sikora, Łukasz Majewski, Andrzej Puszka

**Affiliations:** 1 Department of Technology and Polymer Processing, Faculty of Mechanical Engineering, Lublin University of Technology, Nadbystrzycka 36, 20-618 Lublin, Poland; l.majewski@pollub.pl; 2 Department of Polymer Chemistry, Faculty of Chemistry, Institute of Chemical Sciences, Maria Curie-Skłodowska University in Lublin, ul. Gliniana 33, 20-614 Lublin, Poland; andrzej.puszka@umcs.pl

**Keywords:** processing of plastics, mechanical properties, blown film, optical properties, barrier properties, starch, polylactide, polyethylene

## Abstract

Four different plastics were tested: potato starch based plastic (TPS-P)–BIOPLAST GF 106/02; corn starch based plastic (TPS-C)–BioComp BF 01HP; polylactic acid (polylactide) plastic (PLA)—BioComp BF 7210 and low density polyethylene, trade name Malen E FABS 23-D022; as a petrochemical reference sample. Using the blown film extrusion method and various screw rotational speeds, films were obtained and tested, as a result of which the following were determined: breaking stress, strain at break, static and dynamic friction coefficient of film in longitudinal and transverse direction, puncture resistance and strain at break, color, brightness and gloss of film, surface roughness, barrier properties and microstructure. The biodegradable plastics tested are characterized by comparable or even better mechanical strength than petrochemical polyethylene for the range of film blowing processing parameters used here. The effect of the screw rotational speed on the mechanical characteristics of the films obtained was also demonstrated. With the increase in the screw rotational speed, the decrease of barrier properties was also observed. No correlation between roughness and permeability of gases and water vapor was shown. It was indicated that biodegradable plastics might be competitive for conventional petrochemical materials used in film blowing niche applications where cost, recyclability, optical and water vapor barrier properties are not critical.

## 1. Introduction

Biopolymers produced from renewable sources are a group of materials which are in the center of interest of engineers and processors from many years. This is mainly due to their greatest advantages, i.e., biodegradability and non-toxicity of their biodegradation products, which are desirable features in the current state of environmental pollution. [1,2,3,4]. Biodegradable polymers are considered an attractive alternative and, over time, they will gradually gain more and more applications, especially in the industries involved in the mass production of plastic single-use products, including packaging [5,6]. It is claimed that replacing conventional plastics by biodegradable alternatives is interesting mainly for mass production of products with short lifetime, especially packaging. However, a large group of plastics will be difficult to substitute by biodegradable plastics, especially those applied in civil engineering with long expected lifetime.

Apart from the indisputable advantages, biodegradable polymer materials also have disadvantages which result in limited application possibilities [7,8]. Due to the sensitivity of biodegradable polymers to heat, humidity and shear stresses, they are more difficult to process than the polyolefines [9,10,11]. For these reasons, they may undergo partial thermal and mechanical degradation as early as at the processing stage. In most cases, they also have worse mechanical properties than traditional petrochemical materials, i.e., they are too brittle or rigid, or their tensile strength is too low to successfully replace traditional film materials, for example [12,13]. In addition, in order to be used in the packaging industry for food packaging, they must have appropriate barrier properties, e.g., in relation to oxygen, carbon dioxide and water vapor [14,15,16].

Therefore, numerous research works are conducted on the improvement of properties or limitation of the above-mentioned defects of biodegradable materials. Mechanical, thermal and processing properties of these materials can be modified using various methods. The main ones include the production of composites or nanocomposites with additional components, e.g., fillers, nucleating agents, nano-compounds or fibers [17,18,19,20]. In order to improve the utility properties of materials, processes of their chemical modification may also be used by means of grafting, copolymerization, coating or reactive processing [21,22,23]. Polymer mixtures of two or more components are also known. They have different beneficial properties, and their components cannot meet certain functional requirements individually. Mixtures of various biopolymers (polylactide, polysuccinate, thermoplastic starch, polyvinyl alcohol, chitosan) and mixtures of biopolymers with non-biodegradable polymers, such as polyethylene [24,25,26,27,28], are used in order to obtain compositions with improved performance properties or to improve processing characteristics. The latter allow to achieve good mechanical properties and significantly improve the processing capacity while maintaining compostability [29,30]. Unfortunately, the use of expensive modifications and, in most cases, arduous process of obtaining raw materials for biodegradable polymer production, stringent conditions of processing, packaging, storage and transport of biodegradable materials result in a price multiple times higher than petrochemical polymers, which is one of the main obstacles in their common use [31].

The most important group of bioplastics are biodegradable polymers obtained from renewable raw materials. It is commercially available and is one of the cheaper bioplastics and starch-based plastics [30,32,33]. Biodegradable plastics based on thermoplastic starch may contain almost 100% of the latter [34]. The raw material for their production may be corn starch, potato starch, tapioca starch, cassava starch and rice starch [35,36,37,38,39]. However, it should be modified by assigning its thermoplastic properties by destructuring native starch under strictly defined dynamic and thermal conditions in the extrusion process in the presence of a plasticizer, where under the influence of pressure and high shear stresses the crystalline structure of starch is disrupted and converted to a homogeneous amorphous polymer, capable of thermoplastic processing [40,41,42,43].

Among degradable polymers, polylactide (PLA) plays dominant role. PLA is well degradable only in industrial composting, extremely slow in the environment. PLA and other biodegradable polymers have two main areas of application. The first area is connected with biodegradable polymers produced on a mass scale, and the second area is associated with polymers intended for special applications, mainly in medicine and tissue engineering [5,44,45]. Biodegradable plastics produced on a mass scale are used as packaging materials, orientation films, garden films, thermoforming films, waste bags, trays, cups, bottles, cutlery, disposable products, paper coating material, for printing or in agriculture [46]. Specialized variants are used as bioresorbable implants and surgical threads, clamps, clips, capsules for controlled dosing of medicines, drug carriers, surgical masks, dressings, compresses, clothing for medical personnel, handkerchiefs or cosmetic swabs [47,48,49]. In order to improve the performance of the materials are often used as processes for their chemical modification through grafting, copolymerization, reactive coating or processing. In addition, mixtures of polymers of two or more components are also known. They have different beneficial properties, and their components cannot meet certain functional requirements individually. There may also be mixtures of various biopolymers (polylactide, polysuccinate, thermoplastic starch, polyvinyl alcohol, chitosan), etc.

An extremely important area, which included increasing use of biodegradable polymers is the packaging industry. Plastics, right after paper packaging, are the second most frequently used packaging material in the world. Due to the large amount of waste deposited in landfills, the European Parliament and the Council of the European Union established in December 1994 Directive 94/62/EC regulating waste management in the European Union countries [50]. The aforementioned legal regulation introduced requirements for all types of packaging related to the production and composition of raw materials, suitability for their repeated use and recycling. Unfortunately, recycling of plastic packaging is associated with technical and economic problems, and burning them without energy recovery contributes to environmental pollution. Therefore, there is still ongoing research on the use of natural or biodegradable polymers to produce environmentally friendly packaging. A more detailed description of the biopolymers used for the production of packaging was presented in the previous work [51], while below we present a short description of natural polymers used in food packaging.

Natural polymer films can be divided into protein and polysaccharide films. Among the protein films one can find collagen [52], gelatin [53,54] and casein [55]. The polysaccharide films include starch [56,57,58], chitosan [59,60] and pectin films [61]. The films produced from natural polymers, on the one hand, have good oxygen barrier properties, and on the other hand, they exhibit some disadvantageous mechanical properties, as well as a low water vapor barrier and excessive solubility in a humid environment. These features significantly limit the use of films, therefore, in order to improve their properties, it is necessary to modify polymer components. The most common modifications are with the use of various chemical substances, as well as γ rays [62,63], UV [64,65,66] or temperature [67,68]. The chemical modification of natural polymer film is suitable for plasticizing, the addition of suitable compounds which reduce the intermolecular interactions in the polymer network. The most commonly used plasticizers are glycerol, propylene glycol, polyethylene glycol, sorbitol and sucrose [69,70,71]. Another way to modify films from natural polymers is chemical cross-linking, which can be carried out with synthetic [72] or natural [73] compounds. The appropriate selection of chemical modifications of the film components and the combination of appropriately selected polymers, in addition to improving their functional properties, such as mechanical strength and water barrier, allows to improve the microbiological safety of products and their quality during storage, as well as to extend their storage period.

The previous paper [51] describes the thermal properties (thermogravimetric analysis and differential scanning calorimetry), chemical composition analysis, process of blown film extrusion of biodegradable materials used in the tests and geometric properties of the films obtained in comparison to non-biodegradable material—LDPE. The tests showed that the biodegradable materials used are characterized by much higher film production efficiency than polyethylene, with the same processing parameters. Water has been found in all biodegradable materials, which may hinder the processing process. A higher effect of temperature on bioplastic processability than on polyethylene processing was also determined, which means that uncontrolled temperature fluctuations, e.g., caused by autothermal effect, may significantly interfere with the film extrusion process. The work focuses on correlating the changes in the rotational speed of the extruder screw with the haul-off velocity so that after each change, finally, films with very similar geometric parameters are obtained.

The purpose of this paper is to determine the suitability for production of packaging by way of comparative analysis of mechanical, optical and structural properties of films obtained in the process of blown film extrusion, produced from modern biodegradable and compostable plastics based on polylactide as well as potato and corn starch in relation to film made of conventional low-density polyethylene.

## 2. Experimental

### 2.1. Test Stand

The process of extrusion of tubular film was performed using a MB 45/750 single-screw extruder provided with a standard, traditional screw for LDPE plasticizing and the following specifications: screw diameter D = 45 mm, screw service length L = 28D, manufacturer: Kween B LTD, (Taipei, Taiwan). The extruder is equipped with four ring heaters enabling temperature control on the extrusion head, at the plastic filter and in the feed and metering zones of the plasticizing system, which are additionally paired with fans for temperature stabilization. The screw drive system allowed undisturbed control over the screw rotational speed. The plasticizing system was provided with a cross-wise, spiral core head and an extrusion die with outer diameter of 60 mm and width of 1 mm for blown film extrusion in vertical direction. The internal cylindrical surface of the barrel was smooth along its entire length. The blown film extrusion line featured a collapsing plate and a nip rolls, followed by an idler roll and film windup unit with variable speed control of the windup rollers.

### 2.2. Materials

The blown film extrusion was performed with four different materials: LDPE, trade name Malen E FABS 23-D022, potato starch based plastic (TPS-P)—BIOPLAST GF 106/02, corn starch based plastic (TPS-C)—BioComp BF 01HP, and polylactic acid (polylactide) (PLA)—BioComp BF 7210.

Low density polyethylene Malen E FABS 23-D022 is a typical plastic material for the production of packaging film with a recommended thickness of 20–60 µm. It is used for the production of bags, sacks, heat-shrink films, plastic tubes and films for food packaging. Table 1 presents selected properties of this polyethylene and the film produced from it according to the manufacturer’s data. The processing temperatures recommended by the manufacturer range from 160 °C to 220 °C [74].

BIOPLAST GF 106/02 manufactured by BioTec (Emmerich am Rhein, Germany) with TPS-P is fully biodegradable and fit for composting. It is used primarily for the manufacture of disposable or short-life products, such as food packaging, agricultural films, shopping bags or waste bags. Selected material properties and film made of BIOPLAST GF 106/02 provided by the manufacturer are presented in Table 2. The manufacturer recommends processing using a classic screw for low density polyethylene at temperatures in the range 140–180 °C [75].

TPS-P is a thermoplastic material that does not contain plasticizers and GMO raw materials, but contains natural potato starch [52]. As stated in the literature, BIOPLAST GF 106/02 is a blend of PLA and potato starch [76]. The ATR/FTIR spectra of polymer matrices separated from biodegradable materials are presented in Figure S1 (Supplementary Materials).

BioComp BF 01HP belongs to a family of bioplastics manufactured by MicroTec (Pianiga, Italy) from biodegradable components of organic origin. The material contains polyactic acid (PLA) and corn starch. The primary application is the production of shopping bags. Biodegradation of at least 90% occurs within six months. BioComp BF 01HP is a new material intended primarily for extrusion and blowing of film used to manufacture mainly shopping bags. Selected properties of BioComp BF 01HP according to the manufacturer’s data are provided in Table 3. It is recommended to process using a classic screw for low density polyethylene at temperatures in the range of 150–170 °C [77].

The starch-based materials were designated as TPS-X, where TPS means thermoplastic starch and X represents starch sources (C—Corn starch, P—Potato starch). Due to the fact that both of these materials contain PLA as a matrix for starch, the mechanical properties presented in Table 2 and Table 3 depend mainly on this component. Another factor contributing to differences in the properties of products made of these materials is the ratio of amylose and amylopectin, in both potato as well as corn starches. The amylose content of corn starch is about 25–30%, while the potato starch has an amylose about 20–25% [51].

BioComp BF 7210 manufactured by MicroTec (Pianiga, Italy), just like the previous one, is a plastic primarily intended for film extrusion and can be processed with all conventional blowing lines using standard screw and extruder parameters. The material contains polyactic acid (PLA) and talc. A specially developed formulation provides the material with transparency and makes it a good choice for the production of shopping bags. Selected properties of the material and the film made of it are listed in Table 4. The recommended processing temperatures for BioComp BF 7210 range from 140 to 180 °C [78].

### 2.3. Research Programme and Methodology

In the film extrusion process, the variable factor was the rotational speed of the extruder screw. The assumed values of the analyzed screw rotational speeds were 300, 400 and 500 rpm. Higher screw rotational speed will increase the plastic pressure and shear rate, but the processing machine used was not equipped with plastic pressure and temperature sensors and a screw torque measurement system, which made it impossible to measure these quantities directly. The plasticizing temperatures for each of the plastic material tested were individually selected from the respective manufacturer’s data and proprietary experience of the authors, followed by temperature unification across all heating zones of the extruder. The distribution of temperature values along the length of the plasticizing system was constant for each material, although it differed slightly for individual materials. These temperatures were therefore as follows: LDPE—160 °C, TPS-P—160 °C, TPS-C—155 °C and PLA—145 °C. Other processing parameters corresponding to the geometric characteristics of the obtained film, i.e., the blow-up ratio and haul-off velocity, were set in such a way as to produce a layflat film with the width in the range of 35–36 cm and thickness of a film in the range of 20-µm. The average widths of the film sleeve and the thickness of a film are shown in Table 5. A detailed analysis of the geometric features of the film and the characteristics of the extrusion process can be found in Part I of the paper [51].

Each experimental test was performed in 5 repetitions, and the respective graphs show the mean values with standard deviation bars. Research carried out on the film sleeves produced in the blown film extrusion process included:

(1) Measurements of mechanical properties of the obtained films taking into account the static tensile test, measurement of static and dynamic friction coefficient between two layers of film, and measurement of puncture resistance. The static tensile test in transverse and longitudinal direction was carried out in accordance with the following standards: ISO 527-1, and ISO 527-3 [79,80]. The puncture resistance tests, including the measurement of the film puncture force and strain at break, were carried out in accordance with the recommendations of ASTM D 4649 [81], at the needle advance rate of 10 mm/min. The friction coefficient and the static and dynamic friction force were determined according to ISO 8295 [82] at the set force of 0.2 N and testing speed of 100 mm/min. All these tests were carried out using a ZwickRoell Z010 strength testing machine (Ulm, Germany) with testing speed of 50 mm/min, using dedicated equipment for each test;
(2) Testing of optical properties of film including measurements of color and gloss in CIE L*a*b* system in accordance with ASTM E 308 [83]. In this system, the color is defined on the basis of 3 basic parameters: L (lightness), a (space from red to green) and b (space from yellow to blue). Parameter L is a representation of the grey scale and has values ranging from 0 to 100, where 0 is ideal black and 100 is ideal white. Parameters a and b range from −100 to 100, where the extreme values of these parameters correspond to the maximum color saturation (a = 100—Red, a = −100—Green, b = 100—yellow, b = −100—Blue). The gloss was measured using a comparison method and the measurement was performed in accordance with ISO 2813:2001 [84] at an angle of inclination of 60° of the gap between the light source and the receiver. The tests were performed on a color measurement station equipped with a Ci4200 X-Rite spectrophotometer (Grand Rapids, MI, USA) and dedicated Color iControl software [85];
(3) The roughness of the obtained films was tested using a Contour GT optical profiler manufactured by Bruker (Karlsruhe, Germany), provided with dedicated Vision software and at room temperature. The measurement was carried out for an image with an area of 117.2 × 156.3 µm^2^. Average (*R_a_*) and root-mean-square (*R_q_*) roughness were determined for the tested films. *R_a_* was calculated in accordance with ASME B46.1 [86]. *R_a_* is calculated by an algorithm that measures the average length between the peaks and valleys and the deviation from the mean line on the entire surface within the sampling length. *R_a_* averages all peaks and valleys of the roughness profile and then neutralizes the few outlying points so that the extreme points have no significant impact on the final results. The formula for calculating *R_a_* is given below:
  (1) $$R_{a}=\frac{1}{n}\overset{n}{\underset{i=1}{\sum }}\left|Z_{1}-Z\right|$$
(4) The test of barrier properties of the obtained films was determined on the basis of the water vapor permeability rate, and nitrogen and carbon dioxide permeability. Films obtained at the rotational speed of the extruder screw equal to 300 and 500 rpm were selected for the tests. The water vapor transmission rate was determined in accordance with ISO 15106-1:2003 [87] using the humidity sensor method. The measurements were carried out at a temperature of 38 °C and the permissible relative humidity of the environment ΔRH = 90% using a LYSSY L80-5000 analyzer (Johnsburg, USA). Prior to the measurements, the samples were conditioned at a temperature of 23 °C and humidity of 52%. The permeability of nitrogen and carbon dioxide was determined according to ISO 2556:1 974 [88] using the manometric method. The measurements were performed at a temperature of 23 °C and at a pressure difference of 0.1 MPa using the LYSSY L100-5000 analyzer Johnsburg, USA. Prior to the measurements, the samples were conditioned at a temperature of 23 °C and humidity of 52%;
(5) In order to observe the microstructure of the film samples, a FEI Nova NanoSEM 450 scanning electron microscope (Hillsboro, WA, USA) was used. It is a highly selective SEM operating in high and low vacuum, designed to test the structure and the phase and chemical composition in a wide range of magnifications. For the purpose of observation, a detector of back-scattered electrons for low vacuum (GAD) was used which revealed areas with different chemical composition, and a detector of secondary electrons for low vacuum, operating on the principle of direct detection of electrons (LVD) which visualizes the surface topography.

## 3. Results

### 3.1. Microstructure

**Figure 1:**
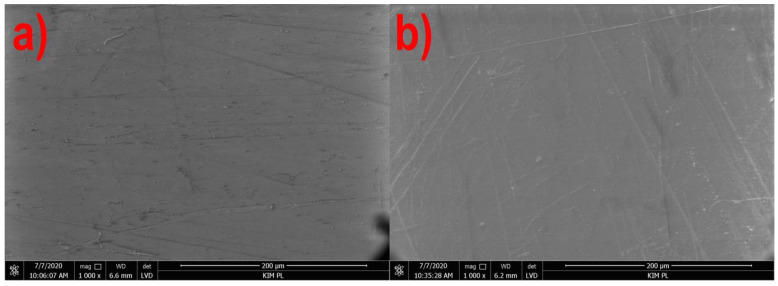
Structure of the formed PE film surface at the rotational speed of the screw: (**a**) 300 rpm, (**b**) 500 rpm.

**Figure 2:**
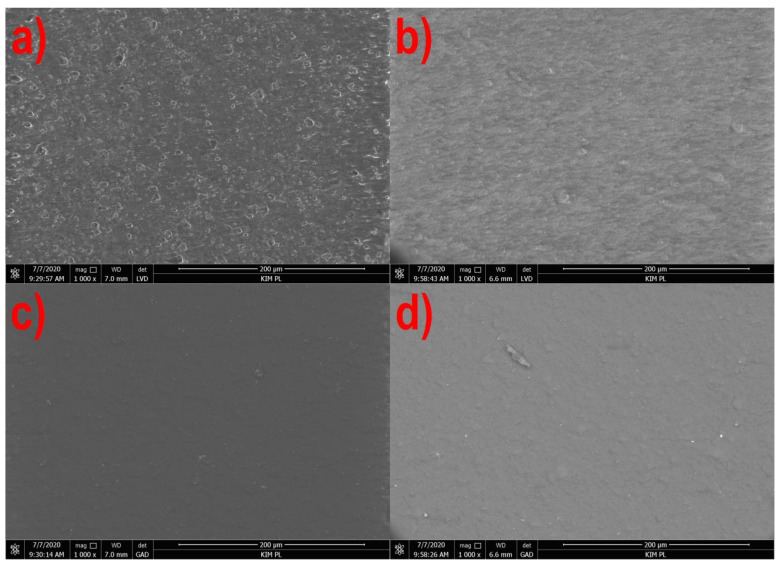
The surface of the PLA film extruded at the screw speed of 300 rpm: (**a**)—LVD, (**c**)—GAD, and 500 rpm (**b**)—LVD, (**d**)—GAD).

**Figure 3:**
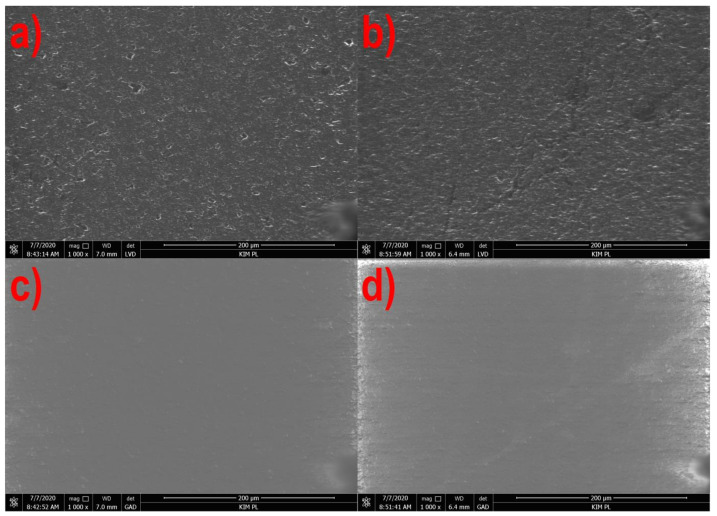
The surface of the TPS-C film extruded at the screw speed of 300 rpm: (**a**)—LVD, (**c**)—GAD and 500 rpm: (**b**)—LVD, (**d**)—GAD.

**Figure 4:**
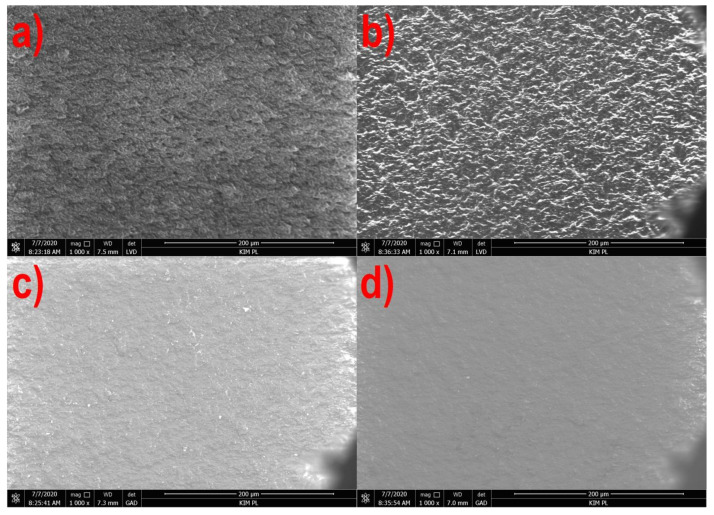
The surface of the TPS-P film extruded at the screw speed of 300 rpm: (**a**)—LVD, (**c**)—GAD and 500 rpm: (**b**)—LVD, (**d**)—GAD).

Figure 1, Figure 2, Figure 3 and Figure 4 show the photographs from the scanning microscope of all four tested films obtained at the rotational speed of the screw equal to 300 and 500 rpm. The observations were always conducted on the outside of the film sleeve. The photographs of the same areas of the samples taken with GAD and LVD were compared. A number of photos of the film surface were used to evaluate the microstructure, whereas the selected films in 1000× magnification are presented below.

Figure 1 shows the topography of the polyethylene film. It is clearly visible that the surface is susceptible to mechanical damage, which is manifested by numerous scratches in the form of long straight lines. The intensity of their occurrence is identical both for the film extruded at the rotational speed of the screw of 300 rpm (Figure 1a) and at the speed of 500 rpm (Figure 1b). However, at a lower extrusion speed, small grains, not visible to the naked eye, appear on the film surface which are not present on the surface of the film extruded at a higher screw rotational speed. These may be unplasticized fragments of the granulate. At a higher speed of the screw in the plasticizing system, the shearing intensity increases, converting mechanical energy of rotary motion into thermal energy and thus improving the efficiency of plasticizing [51]. As expected, the photographs of the PE film from the GAD contained no areas with different chemical composition.

The photographs of the PLA film surface are shown in Figure 2. The LVD revealed numerous irregularities on the surface of the film extruded at a speed of 300 rpm (Figure 2a) which are much less in number on the surface of the film obtained at a higher speed (Figure 2b). Figure 2c,d made using the AD revealed small areas with much higher lightness, i.e., talc grains. Comparing Photographs 2a with 2c, and 2b with 2d, it can be concluded that the location of the grains visible in the GAD corresponds to the location of irregularities observed using the LVD. A significantly concentration of irregularities than talc grains is an evidence of good compatibility of the matrix with the filler which is tightly covered with plastic and only occasionally appears on the surface of the film [89]. The effect of surface smoothing with the increase in the rotational speed of the screw is most probably related to the speed of film output and bulging of the talc grains at lower speeds due to the cooling of the film sleeve before reaching the set blow-up ratio [51].

Figure 3 and Figure 4 show the microscope images of the film surface with corn starch and potato starch, respectively, in a similar manner. In both cases, as for the PE and PLA films, the surface smoothing effect was observed together with increasing the rotational speed of the screw, but the morphology of the film surface from both starches is different.

The surface of the TPS-C film is covered with fine irregularities (Figure 3a,b), but the photographs from the GAD (Figure 3c,d) did not show the presence of phases with different chemical composition. The observed lumps may be non-plasticized residues of the granulate or agglomerates of crystalline starch [10]. Moreover, scratches in the form of dark lines can be observed on the surface of the TPS-C film obtained at the speed of 500 rpm. The morphology of irregularities on the surface of the TPS-P film is not point-like, as in the case of the TPS-C film, but the entire observed surface was characterized by significant roughness (Figure 4a,b). In this case, no phases with different composition were observed (Figure 4c,d) as well, and the obtained image can be determined as typical for a homogeneous amorphous material [90].

### 3.2. Roughness

**Figure 5:**
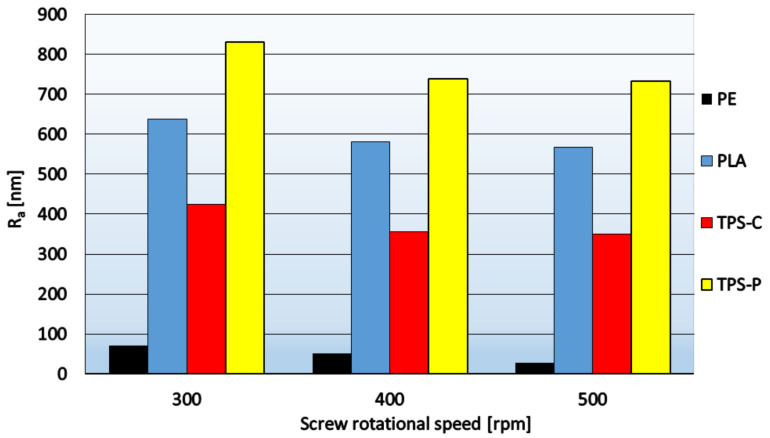
The relationship between parameter *R_a_* and the rotational speed of the extruder screw.

**Figure 6:**
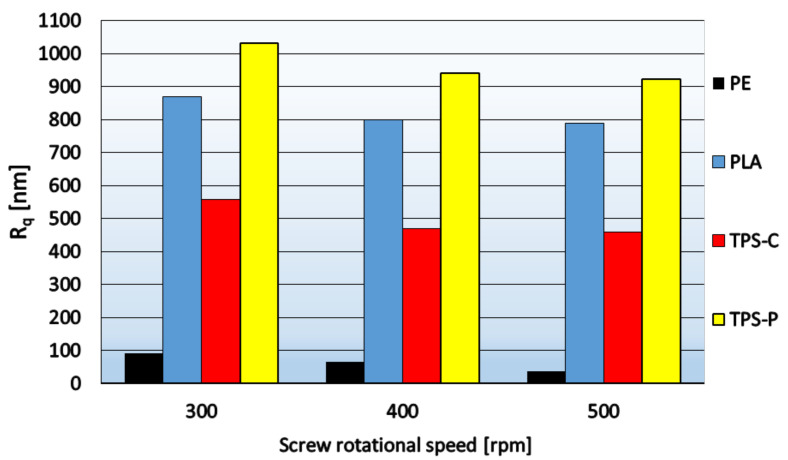
The relationship between parameter *R_q_* and the rotational speed of the extruder screw.

**Figure 7:**
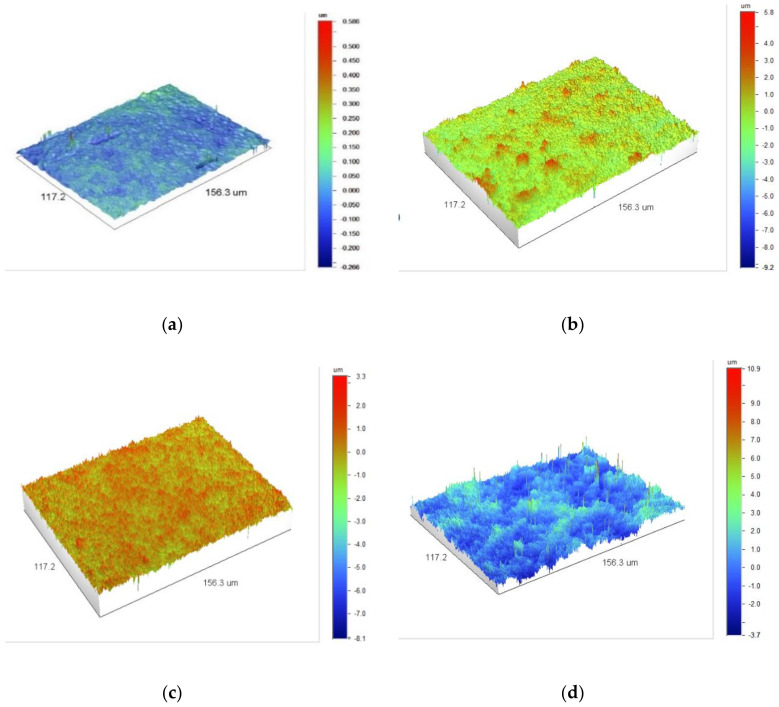
3D images of the topography of the film surface obtained at the screw rotational speed of 400 rpm: (**a**)—PE, (**b**)—PLA, (**c**)—TPS-C, (**d**)—TPS-P.

Two parameters are used to describe the roughness: the arithmetic mean deviation of the profile from the average line *R_a_* and the root mean square roughness *R_q_*. The lower the values of these parameters, the more uniform the film surface. In order to calculate the parameter, the sum between positive and negative values is taken into account, so the phenomenon that the surface is rough may occur, but the integrated value is very low. Therefore, *R_q_* may more accurately reflect the comprehensive characteristics of the surface. Figure 5 and Figure 6 show the relationship between the roughness parameters and the rotational speed of the extruder screw, whereas Figure 7 shows sample 3D images of the film surface topography.

Figure 5 and Figure 6 show that in the case of all materials, as the speed of the extruder screw increases, the film roughness decreases, which is consistent with the obtained microscopic images of the film surface (Figure 1, Figure 2, Figure 3 and Figure 4). Despite its high sensitivity to mechanical damage, the PE film is characterized by several times lower roughness than the film from other materials for all analyzed screw speeds. Some authors associate roughness analysis with the level of crystalline structure of polyethylene. In the analyzed case, it can be observed that the change in the roughness of the PE films is similar to the changes in their crystalline structure [51]. A similar relationship was observed by Sheeja et al. [91]. In the case of the PLA film, its roughness is clearly related to the presence of a filler, which was confirmed by microscopic images (Figure 2) and the photographs taken by the profiler (Figure 7), where spot elevations above the average line were observed. If we analyze materials based on starch, it is clear that the films obtained from materials based on potato starch show much greater roughness. These films also had a 10% higher degree of crystalline structure (from the first heating) as compared to the films obtained from corn starch [51]. This difference may result from the share of amylose and amylopectin contained in individual plastics. This was also noted by Dai et al. [92]. This author stated that a lower content of amylose results in a more uniform film because amylopectin facilitates obtaining a more uniform and compact layer. The roughness results obtained are contrary to these conclusions, and it is difficult to clearly explain this phenomenon. According to the literature, potato starch usually contains less amylose than corn starch [40,51,93,94], but there are also cases in which the authors state that potato starch contains even half more amylose than corn starch [95]. This may be the case in this situation, but the potential cause of differences in the roughness of TPS-C and TPS-P films may also be starch grains which, according to Lindeboom et al., vary from 5 to 30 µm for corn starch and from 5 to 100 µm for potato starch [96].

### 3.3. Friction Coefficient

**Figure 8:**
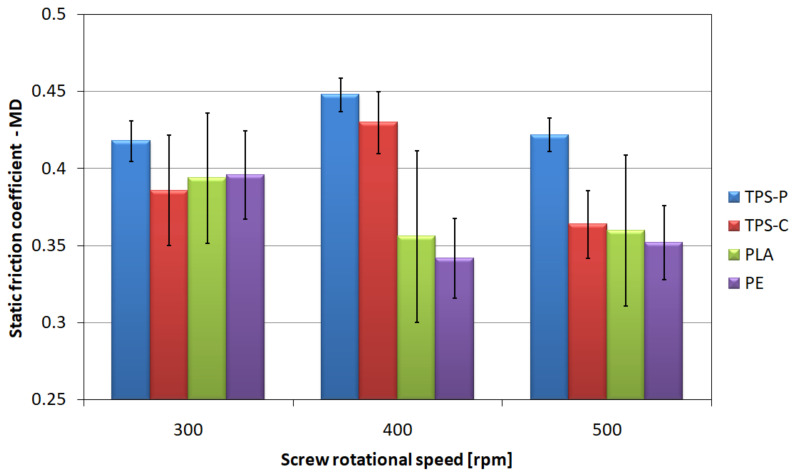
The relationship between the static friction coefficient of the tested films determined in longitudinal direction and the screw rotational speed.

**Figure 9:**
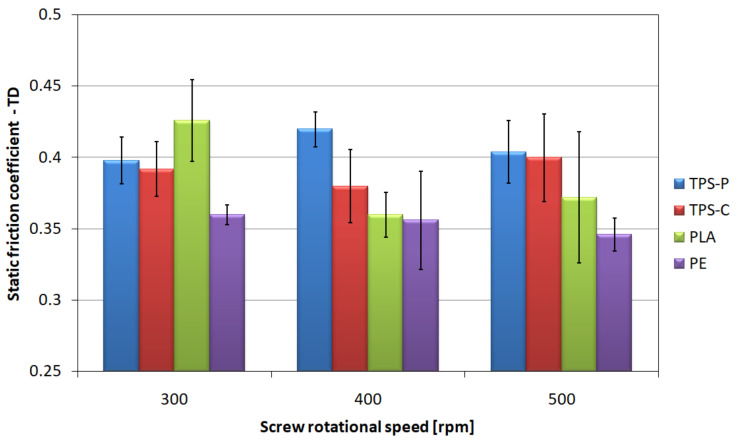
The relationship between the static friction coefficient of the tested films determined in transverse direction and the screw rotational speed.

**Figure 10:**
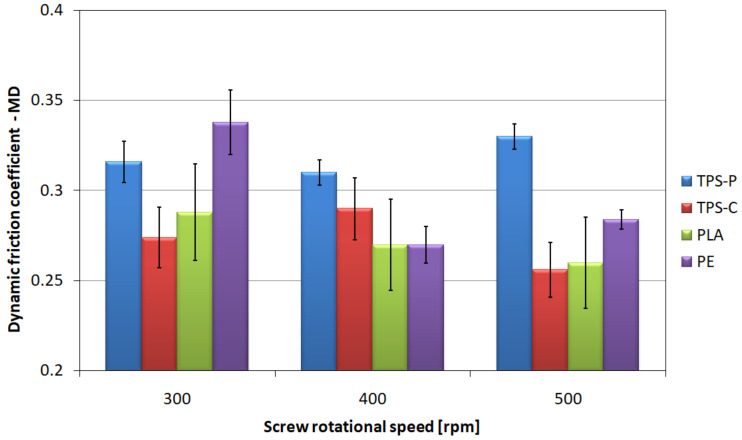
The relationship between the dynamic friction coefficient determined in longitudinal direction and the screw rotational speed.

**Figure 11:**
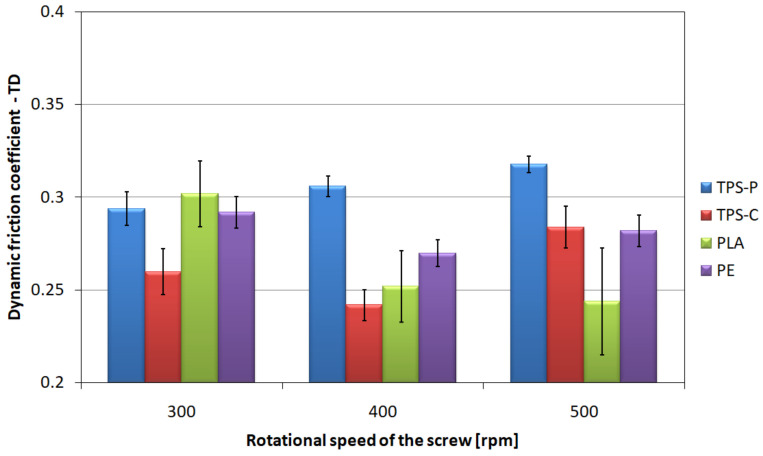
The relationship between the dynamic friction coefficient determined in transverse direction and the screw rotational speed.

The friction coefficient of film layers is an important parameter conditioning the ease of separation and possible use in automatic packaging machines. The values characterizing the friction of two layers of film, i.e., the coefficient of static friction in longitudinal (Figure 8) and transverse (Figure 9) direction, and the coefficient of dynamic friction in longitudinal (Figure 10) and transverse (Figure 11) direction, are characterized by a significant spread of results, which made it difficult to compare individual films with each other and indicate a clear direction of changes in relation to the screw rotational speed, even though the measurements were carried out with care. However, the charts show that the highest mean values of friction coefficients refer to the TPS-P film, and at the same time they indicate a significantly lower spread of results in relation to other films, and the variation coefficients are several times lower. This means that the obtained condition of the TPS-P film surface is repeatable and the friction coefficients remain constant. Previous studies [51] showed that TPS-P had the lowest melt flow index measured at the processing temperature of all tested materials. This means that it had the highest viscosity after leaving the extrusion head, which may be associated with slower stretching of the film in the longitudinal direction and slower cooling that leads to more stable formation of the surface layer. In addition, for all tested screw rotational speeds, the mean values of the TPS-P film friction coefficients in transverse direction were always lower than those obtained during the test performed in longitudinal direction. The PE, TPS-C and PLA films are characterized by significant variation coefficient, which may be a consequence of the susceptibility of these films to damage, such as folds and bends (TPS-C, PLA), scratches (PE) or other processes affecting surface roughness and area of real contact. Although, the mean values suggest that the obtained friction coefficients decrease with the increase in the screw rotational speed, which remains consistent with the obtained microscopic images and the results of the roughness test, changes in the friction coefficients are not obvious. Mainly because the measurement of the friction coefficient is based on the measurement of force, the value of which may depend on many factors. These factors include the area of real contact, adhesion forces responsible for the shear stresses at the contact points which in turn can lead to the elastic deformation of the surface [97]. This is mainly due to the fact that the friction coefficient depends not only on the specific surface area of rubbing surfaces and surface texture but also on the type of material. The literature states that an addition of starch to PLA causes an increase in the coefficient compared to the non-modified PLA, thus reducing the resistance to wear and abrasion [98,99], which is consistent with the obtained values of the friction coefficients. It should also be borne in mind that an increase in the rotational speed of the screw causes an increase in the temperature and pressure of the processed material, which could not be discussed due to the fact that the extruder was not equipped with such sensors.

The friction test in a situation where at least one of the rubbing surfaces is a polymer surface, most often is not continuous. The phenomenon of alternate stopping and moving the rubbing surfaces is then observed, referred to as stick-slip motion. Stopping is observed when the contact area increases, e.g., as a result of local surface deformation, which causes an increase in the strength of adhesive-type interactions. On the other hand, movement occurs when the tangential force reaches value sufficient enough to free the surface from adhesive interactions. Such a phenomenon is observed for polymeric materials for which the glass transition temperatures are much lower than the measurement temperature in which they are in an elastic state—just like the tested materials [51]. Determining the coefficient of friction for materials showing stick-slip motion behavior is burdened with a significant deviation of the results [100,101,102]. In the case of polyethylene, the measured friction force may be influenced also by electrostatic tribocharging phenomenon which causes the tendency of a polyethylene film to adhere to itself or metal parts [103].

### 3.4. Optical Properties

**Figure 12:**
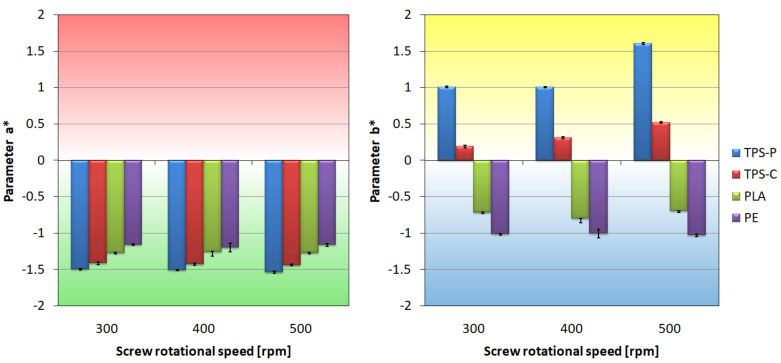
The relationship between the Parameters a* and b* of the material color and the screw rotational speed.

**Figure 13:**
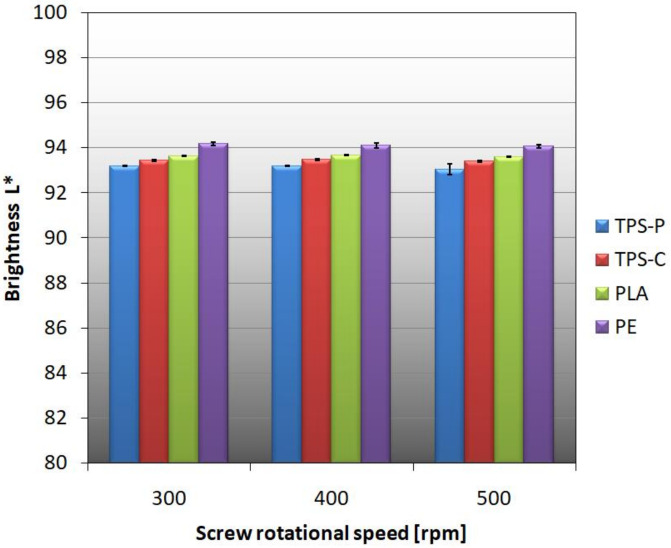
The relationship between the film brightness and the rotational speed of the extruder screw.

Figure 12 shows that the colors of the obtained films are not intense because none of the Parameters a* and b* exceeded the value of 2 or −2, but starch-based films are clearly yellow-tinged in comparison with the PLA and PE films. The analysis of Figure 12 may lead to the conclusion that the screw rotational speed affects the color of the films made of materials containing starch. The Parameter b* for the TPS-C film increases with the increase of the extruder screw rotational speed, and the color changes to more yellow. These changes are small and could potentially be caused by plastic degradation as a result of intensive shearing in the plasticizing system [104]. It can also be assumed that at higher speeds than assumed in the research program, the obtained color changes would be more intense. In the case of the TPS-P film, significant color shift in the direction of yellow occurred only at the screw speed of 500 rpm. High brightness values L of the tested films, shown in Figure 13, point out that the colors are shifted significantly towards white, and the rotational speed does not affect the film lightness.

When analyzing the data collected in Table 6, it can be concluded that the increasing rotational speed of the extruder screw, and consequently the speed of film output and tension, enhances the gloss of the obtained films. For biodegradable films, the increase in gloss is small and not visible to the naked eye. In the case of polyethylene, the increase is noticeable, as between 300 and 400 rpm it is 7%, and between 400 and 500 rpm 13%. However, special attention should be paid to the fact that PE has many times (even up to fifty times) higher gloss than the produced biodegradable films. The gloss is closely related to the roughness, which affects the density of the reflected light and the gloss value [105]. The presented photographs of the film surface and roughness tests explain the gloss test results presented in Table 6.

### 3.5. Tensile Strength

**Figure 14:**
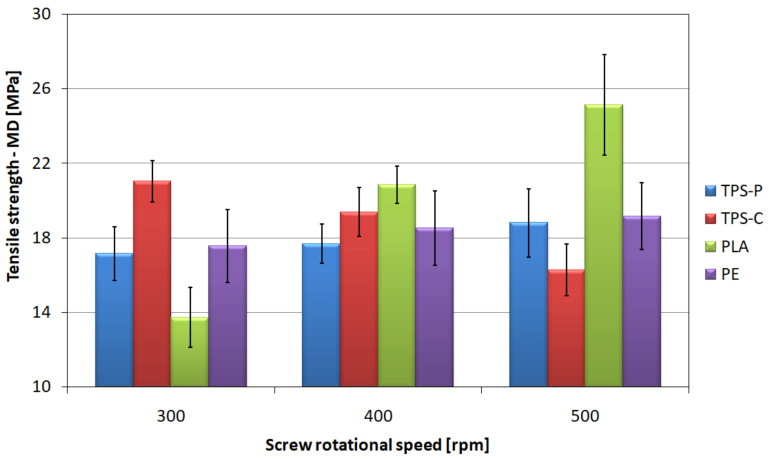
The relationship between the longitudinal tensile strength of the tested films and the screw rotational speed.

With the increase in rotational speed of the extruder screw in the studied range of variability, it was observed that the values of tensile strength of the film in longitudinal direction show an increasing trend in the case of polyethylene, polylactide and plastic based on potato starch, while for the material based on corn starch it decreases (Figure 14). The increase in the screw speed results in the most intensive increase of tensile strength in longitudinal direction for PLA, where the difference between the values of this parameter for extreme screw rotational speeds equals 11.43 MPa. This is an increase in the tensile strength of the tested film by 83.25%, with an increase in the screw speed from 300 to 500 rpm. The values of breaking stress for PE and TPS-P also increase with the increase of the screw rotational speed; however, the observed reinforcements of the films made of these materials are less significant, and for the highest of the tested screw speeds, they are respectively 1.60 MPa and 1.65 MPa, which increases the tensile strength of these films by only 9.1% and 9.6%, respectively, with the screw speed increasing from 300 to 500 rpm. The screw rotational speed shows a significant negative impact on the tensile strength in longitudinal direction of corn starch film. The strength of the TPS-C film at the screw speed of 500 rpm is 4.77 MPa lower than that measured at the speed of 300 rpm, which means a decrease in value by 22.66%. The highest average value of longitudinal tensile strength was recorded for PLA at the highest screw rotational speed and amounted to 25.16 MPa.

**Figure 15:**
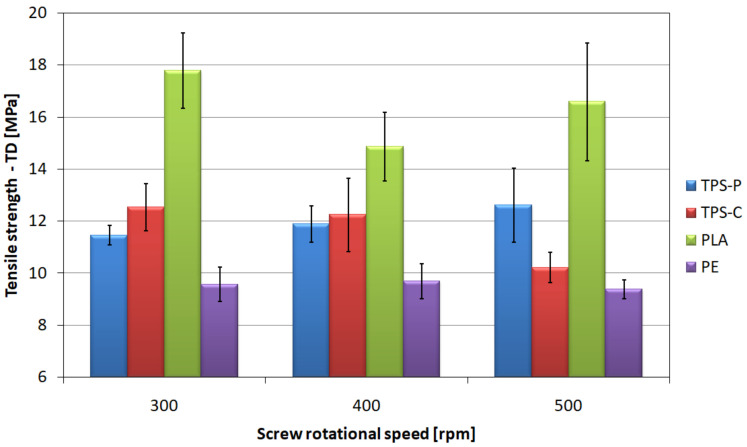
The relationship between the transverse tensile strength of the tested films and the screw rotational speed.

In the case of the relationship between the tensile strength in transverse direction of the extruded film (Figure 15) and the screw rotational speed, only the material based on potato starch shows a slight increase in the average value of the tested magnitude, obtaining 12.62 MPa for the highest speed of the screw.

The PE film does not show any significant changes in the mean value of the tensile strength in transverse direction, which ranges from 9.38 MPa to 9.7 MPa. Taking into account the spread of results, it can be concluded that the rotational speed does not affect the tensile strength in transverse direction of the film made of this material. The effect of the screw rotational speed on the tensile strength of the film based on corn starch, as before, is negative. Although the observed difference in average tensile strength between the speeds of 300 and 400 rpm is small, i.e., 0.3 MPa, but between the average and the highest speed reaches 16.5%. This confirms the negative impact of rotational speed on the strength of the film made of this material. The highest values of tensile strength in transverse direction were obtained for the PLA film; however, a tendency was shown to slightly decrease the average value of this parameter with simultaneous increase of the screw rotational speed, contrary to the strength in longitudinal direction. The highest strength value was recorded for PLA at the speed of 300 rpm and amounted to 17.8 MPa.

It should also be borne in mind that an increase in the rotational speed of the screw causes an increase in the temperature and pressure of the processed material, which could not be discussed due to the fact that the extruder was not equipped with such sensors.

The observed changes in the tensile strength value are mainly related to the phenomenon of the longitudinal orientation of polymer macromolecules. The research plan assumed obtaining blown film with a strictly defined thickness and diameter range, therefore the increase of the extrusion speed was compensated by the appropriately adjusted wind-up roller rotational speed, so that the draw down ratio describing longitudinal stretching remained constant. The increase in the orientation of the polymer macromolecules was achieved by increasing the haul-off velocity of the film, which decreased the cooling efficiency and increased the height of the material freeze line. Thus, the range of the film bubble height, measured from the extrusion head to the freeze line, where the macromolecules were susceptible to orientation under the applied tensile force, increased. Increasing the height of the freeze line has a positive effect on the mechanical properties of the blown film in the longitudinal direction. Therefore, the higher the extrusion speed, the greater the tensile strength in the longitudinal direction. The greatest macromolecules orientation effect was observed for PLA films. The ratio of the tensile strength in the longitudinal direction to the strength in the transverse direction (MD/TD) increased with the rotational speed of the screw, clearly determining the increase in the orientation of macromolecules in the longitudinal direction. The tensile strength in the longitudinal direction and the MD/TD ratio of the PE film and TPS-P also increased with the rotational speed of the screw, but the value change was insignificant. The reason may be the structure of the tested materials. The PLA film has an amorphous structure (degree of crystallinity above 1% [51]), while PE and TPS-P films have a degree of crystallinity of 40–50% [51]. The macromolecules orientation process in partially crystalline materials is different, more complex and depends on many additional factors [106,107,108,109,110,111]. On the other hand, the tensile strength of the TPS-C film decreases in both the longitudinal and transverse directions. This may prove that in TPS-C the phenomena related to mechanical and thermal degradation in the plasticizing system dominate over the strengthening effect of molecular orientation [112,113].

**Figure 16:**
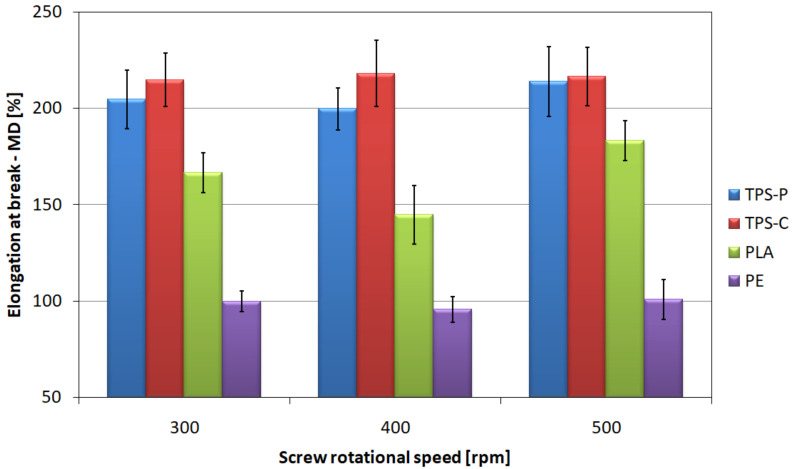
The relationship between the strains at break of the tested films measured in longitudinal direction and the screw rotational speed.

Figure 16 shows the relationship between the elongation at break of the tested films determined in longitudinal direction to the film extrusion direction and the screw rotational speed. On the basis of the graph, it can be concluded that the highest values of strain at break in longitudinal direction for all analyzed screw rotational speeds in longitudinal direction are provided by films made of plastic based on corn starch and potato starch.

The highest average strain for the TPS-P film of 214% was obtained at the speed of 500 rpm, while for the TPS-C film it amounted to 218.3% at 400 rpm. All strains obtained for the films containing starch exceed 200%. Considerably lower mean values of longitudinal strain are shown by the PLA film for which the highest average strain equal to 183.3% was shown for the highest speed of the screw. The drop in strain at the average speed of the screw is probably caused by a slightly lower thickness of the produced film (Table 5). The lowest mean longitudinal strains were obtained for polyethylene film and did not exceed 100%. The effect of the screw rotational speed on the value of these deformations was also not shown. However, some strain measurements obtained for the rotational screw speed of 300 rpm significantly exceeded 100%, hence the larger deviation of the results.

**Figure 17:**
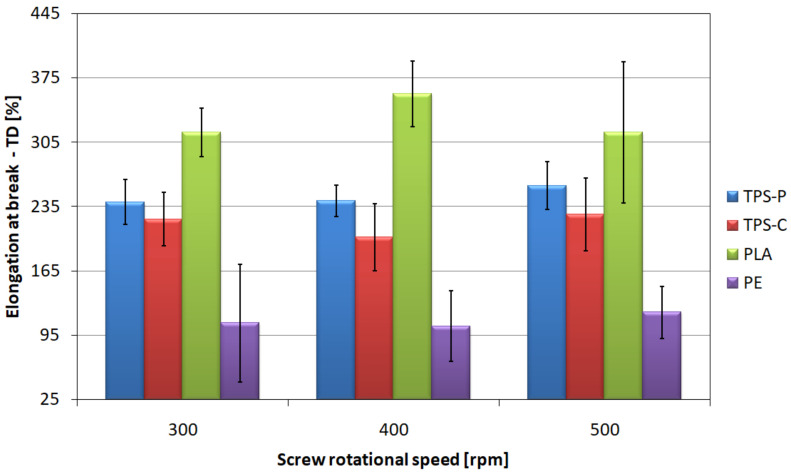
The relationship between the strains at break of the tested films measured in transverse direction and the screw rotational speed.

The results of strain at break measured in transverse direction (Figure 17) are slightly different. When analyzing these relationships, it should be stated that for all materials and rotational speeds of the screw, the observed average values of elongation in transverse direction are higher than those in longitudinal direction. This is caused by a significantly lower degree of elongation in transverse direction during processing [51]. The PLA film is characterized by the highest mean values of strain at break in transverse direction in each of the tested process conditions and they are almost twice as high as the strain in longitudinal direction obtained for this material. The lowest values, similarly to the previous situation, were observed in the PE film, although in this case the obtained strain is also higher in transverse direction than in longitudinal direction by 18.8% for the highest rotational speed.

The observed changes in the mean values of strains at break, both in the longitudinal and transverse directions, were caused by the orientation of the macromolecules in the longitudinal direction. The macromolecules orientation was influenced both by the phenomenon described above, related to the rising of the freeze line, as well as by the specificity of the extrusion blow molding process, where the draw-down ratios of the tested films are several times higher than the blow-up ration [51]. The effect of macromolecules orientation on the strain at break is opposite to that on tensile strength. The orientation of the polymer chains in the longitudinal direction allows for greater deformations in the transverse direction due to their greater mobility in this direction [106,107,109,114].

### 3.6. Puncture Resistance

**Figure 18:**
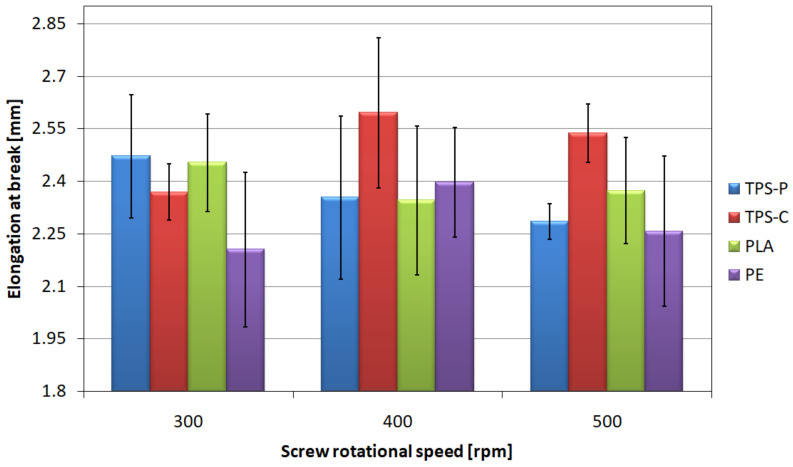
The relationship between the strain at break of the tested films and the screw rotational speed.

The film stretch at the time of puncture (Figure 18) was comparable for all analyzed measurement series, and the mean values of this parameter were between 2.2 and 2.6 mm.

**Figure 19:**
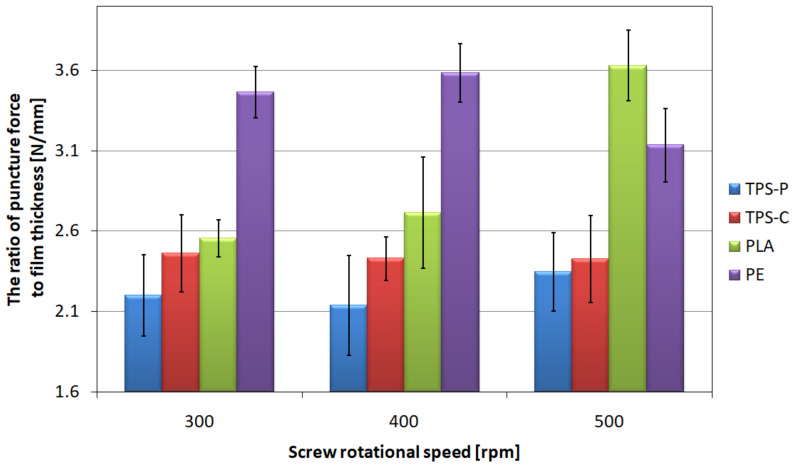
The relationship between the ratios of the force required to break the film and the thickness of the tested films, and the screw rotational speed.

The highest values of puncture resistance (Figure 10.) were obtained for PE—They remain at the level of 3.1–3.6 N/mm for all analyzed rotational speeds of the screw. Therefore, it can be concluded that the rotational speed of the extruder screw does not significantly affect the puncture resistance of PE. In the case of PLA, an increase in the puncture resistance was observed together with an increase in the screw rotational speed of the screw. The increase between 300 rpm and 400 rpm is small, but it is as high as 46% between 300 rpm and 500 rpm. As a result, it can be stated that an increase in the screw rotational speed has a positive effect on the puncture resistance of the PLA film. On the basis of Figure 19, it can also be concluded that the screw rotational speed does not affect the puncture resistance of the TPS-C film the average value of which ranges from 2.42 N/mm to 2.46 N/mm, especially since the results for these series of measurements were characterized by the lowest variation of values of this parameter. For the TPS-P film, a slight difference in average values was obtained, equal to 6% between the speeds of 300 rpm and 500 rpm; however, significant standard deviations do not allow for clear determination of the direction of changes in the puncture resistance.

The specificity of the puncture resistance measurement makes it a parameter that depends to a large extent on the orientation of the polymer macromolecules. The puncture tip applies force in the middle of a certain surface causing the sample to be stretched in the longitudinal and transverse directions simultaneously. Therefore, a positive influence of the screw rotational speed on the puncture resistance of PLA film was observed, because by analyzing the results obtained in the static tensile test, the orientation of the macromolecules of this material in the longitudinal direction was proved. However values of this parameter depend also on the local film thickness, length of polymer chains, force of interaction between the polymer macromolecules and locally occurring material defects, as well as the location of filler particles [106,115]. The highest values of puncture resistance observed for PE film may be dictated by the composition of the material, as the polyethylene used in the research is a basically one-component composition containing only trace amounts of auxiliary substances [74].

### 3.7. Barrier Properties

**Figure 20:**
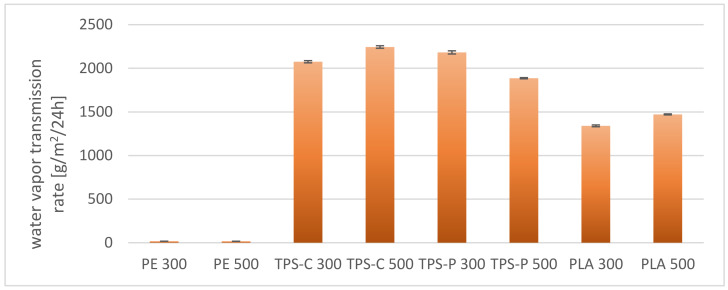
Water vapor transmission rate of selected films.

**Figure 21:**
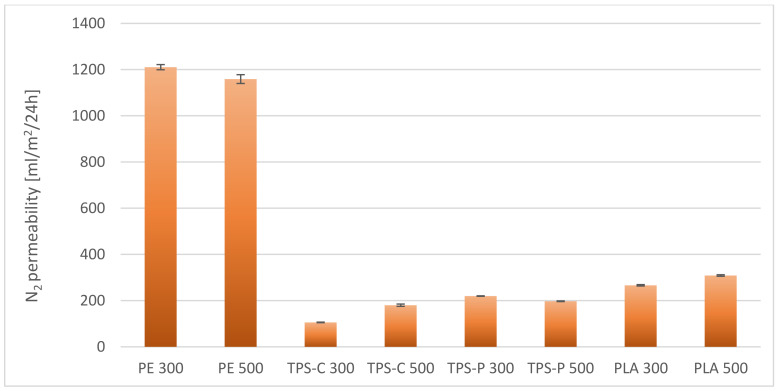
Nitrogen permeability through selected films.

**Figure 22:**
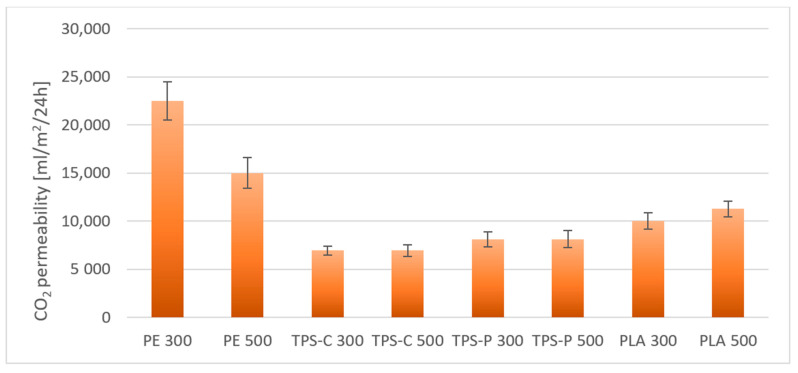
Carbon dioxide permeability through selected films.

As shown in Figure 20, the most favorable water vapor transmittance rate are shown by films obtained from PE, the lower—From thermoplastic starch. Polyethylene is known to have poor water vapor permeability due to the hydrophobic nature of macromolecules [116]. On the other hand, starch-based films are very permeable to water vapor due to their hydrophilicity. An additional factor affecting the foregoing is the presence of plasticizers added during the modification of native starch in order to produce thermoplastic starch and enhancing the hydrophilic nature of the finished material [117,118]. In addition, in the case of starch-based plastics, the amylose content and the degree of crystallinity are also important, as they limit moisture transmission as their values increase [119]. Lower water vapor permeability values obtained for the PLA film indicate lower hydrophilisation of this material compared to starch. The presence of a filler in the form of talc also decreases the permeability of water vapor and other gases through the films by higher tortuosity. Literature data show that the effect of low talc content is negligible for PLA permeability, but the content of a dozen or so percent effectively limits gas transmission by increasing the degree of crystallinity. It is consistent with the free volume theory, where the increase in crystallinity reduces the number of amorphous areas, which are definitely more susceptible to gas permeation [120,121]. Different relationships were observed in the case of nitrogen and carbon dioxide permeability through the obtained films. As shown in Figure 21 and Figure 22, the lowest nitrogen and carbon dioxide permeability were demonstrated by films obtained from PE, while the highest ones—from films obtained from biodegradable materials. Nevertheless, among the films obtained from bio-plastics, the best results were obtained for starch-based films. Generally, for bio-plastics, increasing the rotational speed of the extruder screw caused an increase in the permeability of both nitrogen and carbon dioxide (except for the TPS-P films). In the case of the PE film, increasing the speed of the extruder screw resulted in decreasing the permeability of nitrogen and carbon dioxide.

In the literature, one can find correlations between the chemical structure, morphological properties such as density, degree of crystallinity, roughness or orientation of macromolecules, and barrier properties [122]. In the case of biodegradable plastics, the water content also has a significant impact, which negatively affects their permeability by activating the gas absorption due to the increased ability to dissolve and diffuse through the matrix [123]. Comparing the obtained results of the surface roughness of the film and the obtained degrees of crystallinity and moisture content [51] with the barrier properties, it can be concluded that there is no close correlation between these parameters and the permeability of gases and water vapor. It should also be borne in mind that an increase in the rotational speed of the screw causes an increase in the temperature and pressure of the processed material, which could not be discussed due to the fact that the extruder was not equipped with such sensors.

## 4. Conclusions

The tests conducted have shown that modern compostable and biodegradable materials in many respects may be competitive for conventional petrochemical materials used for blown film extrusion. The TPS-P, TPS-C and PLA films showed comparable strength properties in relation to the PE film, or even better, especially in transverse direction to the extrusion direction. However, polyethylene proved to be much more resistant to puncture than starch-based plastics.

The screw rotational speed does not affect the strength of the polyethylene film and this is confirmed by the large processing window of this material. The screw rotational speed has a slight impact on the strength characteristics of the TPS-P film and significantly affects the strength of the TPS-C and PLA films. The screw rotational speed has a negative impact on the mechanical properties of the material based on corn starch. The color tests showed the shift of the TPS-C film color towards yellow, which can be interpreted as degradation of the material by intensive shearing in the plasticizing system and may constitute a potential cause of deterioration of mechanical properties. In the case of the PLA film, a favorable effect of the screw rotational speed was observed both on its tensile strength and puncture resistance. Even a double increase in strength parameters at extreme speeds was pointed out. Therefore, it is recommended to extrude the PLA film at high screw rotational speed.

The tests of tribological properties revealed the highest values of friction coefficients and the smallest spread of results for the TPS-P film. This means higher repeatability of the obtained surface condition than for other materials which may be more susceptible to surface damage, bending and scratches. The profiler film test results confirm this observation, as the TPS-P films had the highest values of roughness parameters.

Optical tests confirm that the PE film has better optical properties compared to biodegradable films, the gloss of the PE film is several dozen times higher, and the change in color is not observed as the screw speed increases.

The film surface roughness depends on the screw speed. As this parameter increases, the film surface roughness decreases. The highest roughness is assigned to biodegradable films and the smallest roughness—To the PE film, which correlates with the microscopic structure of the film surface.

The PE film has the most beneficial barrier properties in relation to water vapor, and the film made of thermoplastic starch has the worst barrier properties, whereas in the case of nitrogen and carbon dioxide permeability it is exactly the opposite.

Due to comparable mechanical strength, all three tested compostable materials can successfully replace PE film in the production of disposable film packaging elements intended for short-term use. Examples of such items are disposable shopping bags, bags for vegetables and fruit, and disposable ready-made food containers. These types of products made of petrochemical materials are responsible for significant environmental pollution because their production run is very big and their lifetime is short. The ability to decompose TPS-C, TPS-P and PLA films allows to eliminate this problem. In addition, comparable coefficients of friction of the above-mentioned biomaterials (compared to PE) will allow the use of the same packaging machines. In contrast, the ability to transmit water vapor can help to reduce the build-up of water vapor when packing products with a high moisture content, such as fruit.

## Figures and Tables

**Table 1:** Selected properties of low density polyethylene Malen E FABS 23-D022 [74].

Property	Value	Unit
Density	923	kg/m^3^
MFR (190 °C/2.16 kg)	1.95	g/10 min
Vicat softening temperature (50 °C/h, 10 N)	91	°C
Melting temperature	112	°C
Content of polymer anti-blocking	0.017	%
Slip agent content (oleamide)	0.05	%
Tensile strength in longitudinal direction *	18	MPa
Tensile strength in transverse direction *	17	MPa
Strain at break in longitudinal direction *	450	%
Strain at break in transverse direction *	540	%
Puncture resistance (EN ISO 7765-1) *	190	G
Haze (ASTM D 1003) *	8	%

* The values were determined for a film with a thickness of 50 µm, extruded at a temperature of 180 °C and a blow-up ratio of 1:2.5.

**Table 2:** Selected properties of BIOPLAST GF 106/02 [75].

Property	Value	Unit
Size of granulate	1.5–2.5	mm
Melting temperature	120–130	°C
Density	1200–1300	kg/m^3^
Bulk density	740–800	kg/m^3^
MFI (190 °C/5 kg)	2.5–5.5	g/10 min
Water content	<0.5	wt%
Tensile strength in longitudinal direction *	20–35	MPa
Tensile strength in transverse direction *	20–35	MPa
Strain at break in longitudinal direction *	600–900	%
Strain at break in transverse direction *	600–900	%
Oxygen permeability (80 µm)	~750	cm^3^/(m^2^ d bar)
Water vapor permeability (80 µm)	~120	g/(m^2^ d)

* The specified mechanical properties were determined for a film with a thickness of 10 µm.

**Table 3:** Selected properties of BioComp BF 01HP [77].

Property	Value	Unit
Density	1270–1300	kg/m^3^
MFI (190 °C/2.16 kg)	2–6	g/10 min
Moisture content	0.07–0.09	wt%
Melting temperature	110–130	°C
Tensile strength in longitudinal direction *	18	MPa
Tensile strength in transverse direction *	10	MPa
Strain at break in longitudinal direction *	200	%
Strain at break in transverse direction *	250	%
Longitudinal tear resistance (Elmendorf) *	1050	mN
Transverse tear resistance (Elmendorf) *	150	mN
Puncture resistance (EN ISO 7765-1) *	180	g

* The specified values of mechanical properties are given for film with a thickness of 10 µm.

**Table 4:** Selected properties of BioComp BF 7210 [78].

Property	Value	Unit
Density	1380	kg/m^3^
MFI (190 °C/5 kg)	10.76	g/10 min
Moisture content	0.15–0.18	wt%
Melting temperature	140–150	°C
Tensile strength in longitudinal direction *	35.7	MPa
Tensile strength in transverse direction *	25.7	MPa
Strain at break in longitudinal direction *	250	%
Strain at break in transverse direction *	610	%
Longitudinal tear resistance (Elmendorf) *	400	mN
Transverse tear resistance (Elmendorf) *	2300	mN

* The specified values of mechanical properties are given for film with a thickness of 10 µm.

**Table 5:** Geometrical features of the tested film sleeves [51].

Material	Screw rpm	Width of a Film (cm)	Film Thickness (µm)
TPS-P	300	35.3 ± 0.4	25.6 ± 1.7
	400	35.8 ± 0.3	27.6 ± 2.8
	500	36.2 ± 0.1	25.8 ± 1.4
TPS-C	300	35.5 ± 0.2	26.6 ± 1.1
	400	35.7 ± 0.1	25.0 ± 1.0
	500	36.2 ± 0.2	24.2 ± 1.5
PLA	300	35.2 ± 0.2	25.4 ± 1.5
	400	35.5 ± 0.2	20.6 ± 1.3
	500	36.5 ± 0.2	24.2 ± 1.8
PE	300	36.1 ± 0.1	26.2 ± 1.3
	400	36.0 ± 0.1	24.0 ± 0.7
	500	36.4 ± 0.1	27.2 ± 0.8

**Table 6:** The relationship between the average gloss values of the obtained films and the rotational speed of the extruder screw. Standard deviation values are given in parentheses.

Screw Rotational Speed (rpm)	Gloss		
	300	400	500
TPS-P	5.135 (0.04)	5.012 (0.14)	5.71 (0.3)
TPS-C	5.432 (0.14)	5.642 (0.29)	6.067 (0.21)
PLA	5.810 (0.3)	6.360 (0.51)	6.362 (0.31
PE	242.940 (8.29)	260.977 (7.54)	298.695 (13.42)

## Data Availability

The data presented in this study are available on request from the corresponding author.

## Supplementary Materials

The following are available online at https://www.mdpi.com/article/10.3390/ma14102523/s1, Figure S1: FTIR/ATR spectra of obtained polymer matrices.
