# Supplementary material for: Modern Biodegradable Plastics—Processing and Properties Part II
Source: Materials (Basel). 2021 May 12;14(10):2523. doi: 10.3390/ma14102523 (PMC8152069; doi:10.3390/ma14102523)
Supplement: Supplementary file 1 [file materials-14-02523-s001.zip › materials-1190870-supplementary.pdf]

materials

Supplementary Materials

Modern Biodegradable Plastics—Processing and Properties Part II

Janusz W. Sikora [1,*], Łukasz Majewski [1] and Andrzej Puszka [2]

[1] Department of Technology and Polymer Processing, Faculty of Mechanical Engineering, Lublin University of Technology, Nadbystrzycka 36, 20-618 Lublin, Poland; l.majewski@pollub.pl
[2] Department of Polymer Chemistry, Institute of Chemical Sciences, Faculty of Chemistry, Maria Curie-Skłodowska University in Lublin, ul. Gliniana 33, 20-614 Lublin, Poland; andrzej.puszka@umcs.pl
* Correspondence: janusz.sikora@pollub.pl; Tel.: +48-8153-84-222

Citation: Sikora, J.W.; Majewski, Ł.; Puszka, A. Modern Biodegradable Plastics—Processing and Properties Part II. *Materials* **2021**, *14*, 2523. https://doi.org/10.3390/ma14102523

Academic Editors: Andrea Sorrentino and Loic Hilliou

Received: 3 April 2021
Accepted: 10 May 2021
Published: 12 May 2021

Publisher's Note: MDPI stays neutral with regard to jurisdictional claims in published maps and institutional affiliations.

Copyright: © 2021 by the authors. Licensee MDPI, Basel, Switzerland. This article is an open access article distributed under the terms and conditions of the Creative Commons Attribution (CC BY) license (http://creativecommons.org/licenses/by/4.0/).

1. Determination of the Polymer Matrix in the Material

1.1. Procedure

3 g of granules were weighed into a Falcon tube and 30 ml of chloroform were added. After dissolving the material, the precipitate was centrifuged and the solution from above the precipitate was decanted. Then 20ml of methanol was added to the solution. The precipitated polymer was filtered under reduced pressure and transferred to a Petri dish and dried. A FTIR / ATR spectrum was obtained for the dried polymer.

1.2. Analysis

The attenuated total reflection (ATR) was recorded based on Fourier transform infrared (ATR/FT-IR) spectroscopy using a TENSOR 27 Bruker spectrometer equipped with a diamond crystal (Ettlingen, Germany). The spectra were recorded in the range of 4000–600cm^{-1} with 32 scans per spectrum at a resolution of 4 cm^{-1}.

1.3. Results

The obtained FTIR/ATR spectra are shown in Figure S1. As can be seen, the FTIR/ATR spectra of all samples are almost identical, which indicates that the same polymer matrices were tested. The presence of absorption bands at about 3360 cm^{-1} (connected with stretching vibrations of –OH groups) may be due to incomplete drying of the material (presence of methanol) and/or the presence of a small amount of starch that may not have been drained sufficiently during preparation (first step before adding of methanol).

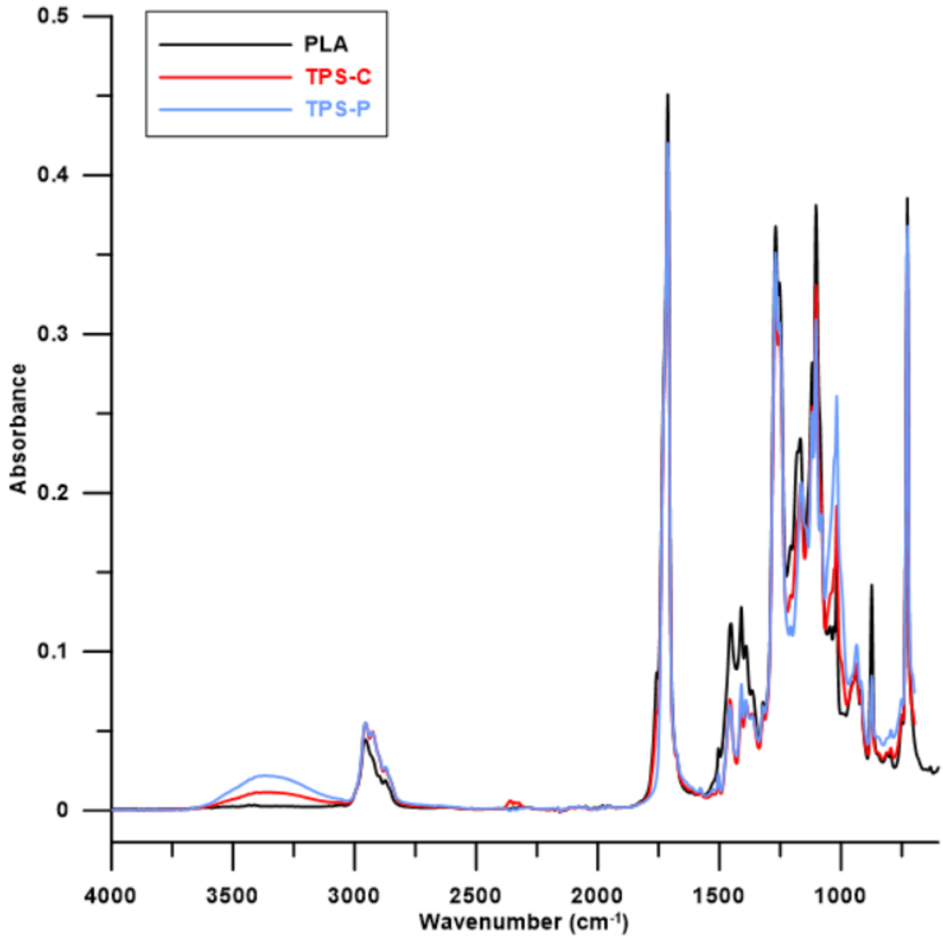

Figure S1. FTIR/ATR spectra of obtained polymer matrices.
